# Mitochondrial and Autophagic Regulation of Adult Neurogenesis in the Healthy and Diseased Brain

**DOI:** 10.3390/ijms22073342

**Published:** 2021-03-24

**Authors:** Hansruedi Büeler

**Affiliations:** School of Life Sciences and Technology, Harbin Institute of Technology, Harbin 150080, China; hbueler@hit.edu.cn

**Keywords:** adult neurogenesis, hippocampus, mitochondrial metabolism, mitochondrial dynamics, reactive oxygen species (ROS), autophagy/mitophagy, neurodegeneration, cognitive dysfunction, psychological stress, mood disorders

## Abstract

Adult neurogenesis is a highly regulated process during which new neurons are generated from neural stem cells in two discrete regions of the adult brain: the subventricular zone of the lateral ventricle and the subgranular zone of the dentate gyrus in the hippocampus. Defects of adult hippocampal neurogenesis have been linked to cognitive decline and dysfunction during natural aging and in neurodegenerative diseases, as well as psychological stress-induced mood disorders. Understanding the mechanisms and pathways that regulate adult neurogenesis is crucial to improving preventative measures and therapies for these conditions. Accumulating evidence shows that mitochondria directly regulate various steps and phases of adult neurogenesis. This review summarizes recent findings on how mitochondrial metabolism, dynamics, and reactive oxygen species control several aspects of adult neural stem cell function and their differentiation to newborn neurons. It also discusses the importance of autophagy for adult neurogenesis, and how mitochondrial and autophagic dysfunction may contribute to cognitive defects and stress-induced mood disorders by compromising adult neurogenesis. Finally, I suggest possible ways to target mitochondrial function as a strategy for stem cell-based interventions and treatments for cognitive and mood disorders.

## 1. Introduction

Neurogenesis produces new neurons from neural stem cells (NSC) and is essential for brain development. In addition, NSC and neurogenesis occur in two discrete regions of the adult brain: the subventricular zone (SVZ) of the lateral ventricle and the subgranular zone (SGZ) of dentate gyrus (DG) in the hippocampus [1]. In these two areas, radial glia-like NSC self-renew or give rise to transiently proliferating intermediate progenitor cells (IPC) that subsequently form neuroblasts. In the SVZ, neuroblasts migrate along the rostral migratory stream to reach the olfactory bulb (OB), where newborn neurons mature and form synapses with existing olfactory sensory neurons. Functional integration of newborn neurons adds plasticity to the OB circuitry and is important for olfactory discrimination, learning, and memory [2,3]. In the hippocampus, neuroblasts migrate into the granule cell layer, where they differentiate into mature neurons that integrate into the existing DG circuitry [4]. Adult hippocampal neurogenesis (AHN) is important for spatial and contextual pattern separation, which requires the DG [5,6] and depends on immature, adult-born granule neurons that display increased excitability and plasticity [7,8,9]. Blocking neurogenesis in mice by focal X-ray irradiation reduced spatial learning and memory performance, but only when the cues were presented with little spatial separation [10]. Likewise, conditional ablation of the GDNF receptor GFRα1, which inhibited dendritic maturation of immature DG granule cells, impaired the ability of mice to distinguish between identical objects placed in similar, but not dissimilar, locations [11]. Several other groups confirmed that deficits of AHN compromise pattern separation [12,13,14,15]. In contrast, promoting AHN through moderate exercise [16,17] or by inducible genetic expansion of newborn neurons [18] improved performance in spatial pattern separation tasks.

There is some controversy as to whether adult neurogenesis is maintained throughout life in humans or ends between childhood and early adult life. Several groups detected proliferating NSC as well as immature and mature newborn neurons in the DG even in aged humans [19,20,21]. However, other groups have argued that hippocampal neurogenesis declines sharply in children and becomes undetectable in adults [22]. Time translation studies that allow for cross-species comparisons showed that hippocampal neurogenesis is a continuous process that starts during embryonic brain development [23] and plateaus out at a low level in all species as they age, and it has been suggested that even low levels of neurogenesis in adult humans could be sufficient to affect certain behaviors and mitigate age-dependent cognitive defects [24,25]. The discrepancy between two recent, seemingly similar studies [20,22] may be explained by differences in sampling postmortem brain tissue, different methods for cell quantification, and interpretation of cell types, or a combination thereof. Collectively, the studies highlight the need for standardized procedures and better markers for neurogenesis when working with postmortem tissue [26,27,28,29].

## 2. Metabolic Regulation of Stem Cell Maintenance versus Differentiation

We have a considerable understanding of the extracellular cues (e.g., morphogens, neurotrophic and growth factors, cytokines) and intracellular signaling pathways (e.g., Notch and Wnt signaling, transcription factors, miRNAs, cell cycle regulators) that control adult NSC maintenance, activation, proliferation, and neuronal differentiation [30,31]. In contrast, the regulatory function of mitochondria and cell metabolism during adult neurogenesis has only recently been illuminated.

Mitochondrial function and dynamics are crucial for the control of cell proliferation and differentiation [32,33,34]. Stem cells either self-renew to maintain stemness or differentiate into tissue-specific cells, and the balance between self-renewal and differentiation is regulated by dynamic changes in cell metabolism. In the hematopoietic system and the heart, stemness is associated with glycolysis [35,36,37,38]. In contrast, differentiation of stem cells into mature cells requires a switch from glycolysis to increased mitochondrial respiration to satisfy the increased energy demand of differentiated cells [35,36,38,39,40]. Likewise, differentiation of induced pluripotent stem cells (iPSC) involves remodeling of the mitochondrial network for increased mitochondrial activity, while reprogramming of somatic cells to iPSC demands an opposite transition from mitochondrial oxidative phosphorylation (OXPHOS) to glycolysis [41,42,43].

In iPSC-derived NPC, the switch from glycolysis to OXPHOS is characterized by downregulation of glycolytic enzymes hexokinase (HK2) and lactate dehydrogenase (LDHA) and increased expression of pyruvate kinase 1 [44]. Constitutive overexpression of HK2 and LDHA during NPC differentiation caused neuronal death, showing that shutting off glycolysis to promote OXPHOS is a prerequisite for neuronal differentiation of iPSC [44]. Early indications that cell metabolism is also crucial for adult neurogenesis came from the observation that a diet enriched in poly-unsaturated fatty acids induced neurogenesis in the SVZ and the DG of adult mice [45]. Later, mice with a conditional ablation of fatty acid synthase in NSPC provided direct confirmation that lipid metabolism regulated adult neurogenesis [46], and this has since been corroborated by several other studies [47,48,49]. NSPC sustained aerobic respiration by expressing enzymes required for fatty acid oxidation (FAO), which increased their proliferation [50]. In addition, FAO by carnitine palmitoyltransferase 1a (Cpt1a) was high in qNSC but reduced in proliferating NSPC, suggesting that a high rate of FAO is required for NSC maintenance [51]. An in-depth review on how lipids regulate NSC function has recently been published [52].

**Figure 1:**
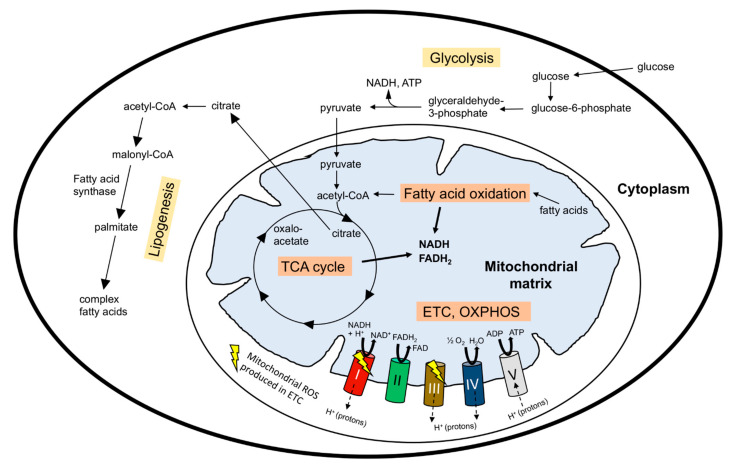
Simplified depiction of the cellular metabolic pathways discussed in this review. Glycolysis converts glucose into pyruvate that is transported into mitochondria and converted to acetyl-CoA. Acetyl-CoA is also produced during fatty acid oxidation (FAO) in the mitochondrial matrix. Acetyl-CoA enters the tricarboxylic acid (TCA) cycle that produces the substrates NADH and FADH_2_ (for respiratory complex I and II, respectively) required for oxidative phosphorylation (OXPHOS). Electron transport during respiration is coupled to proton (H^+^) export from the mitochondrial matrix to the inter-membrane space. This generates an electrochemical gradient Δψm where the H^+^ concentration is higher in the inter-membrane space than in the matrix. When H^+^ flow back into the matrix through ATP synthase (complex V), the energy of this gradient is used to produce ATP. During OXPHOS, electrons leak at respiratory complex I and III and react with molecular oxygen to produce ROS (superoxide, which is converted to H_2_O_2_). In addition to the TCA cycle, NADH and FADH_2_ are produced during FAO. Citrate produced in the TCA cycle exits mitochondria and is converted back to acetyl-CoA in the cytoplasm, where it is used to generate complex fatty acids through de novo lipogenesis. For FAO, complex fatty acids must be transported into the mitochondrial matrix via a carnitine shuttle system and two distinct transporters located in the outer and inner mitochondrial membrane. For simplicity, transporter proteins for various molecules are not shown in the figure. Arrows with dashes indicate the direction of proton (H^+^) transport/flow between the mitochondrial matrix and the intermembrane space.

Collectively, these studies showed that stem cells rely primarily on glycolysis and FAO, and that a progressive transition to OXPHOS is required for the differentiation of stem cells of many, if not all, tissues. Figure 1 depicts a simplified scheme of the cell metabolic pathways discussed in this review.

## 3. Mitochondria Regulate Adult Neural Stem Cell Function and Neurogenesis

Mitochondria not only produce ATP, but also act as cellular signaling hubs through the release of metabolites, reactive oxygen species (ROS), and proteins. As such, mitochondria directly influence cytosolic signaling pathways and regulate nuclear gene transcription via mitochondria-to-nucleus retrograde communication and epigenetic gene modifications [53]. In addition, mitochondria undergo dynamic morphological changes through fission and fusion events, which are referred to as mitochondrial dynamics [34]. Recent work has shown that mitochondrial metabolism, ROS signaling, and dynamics all have crucial functions for NSPC and throughout adult neurogenesis. Although I discuss these topics in separate paragraphs below, they are closely and functionally connected. For example, mitochondrial fission is linked to ROS production and a prerequisite for mitophagy that degrades depolarized and aged mitochondria, whereas fusion is required to maintain a functional mitochondrial population in cells by enabling the exchange of contents between mitochondria, thereby allowing damaged mitochondria to regain lost essential components such as mitochondrial DNA [34,54,55].

### 3.1. Mitochondrial Metabolism-Regulated Adult Neurogenesis

Single-cell RNA sequencing revealed that quiescent NSC (qNSC) in the adult hippocampus rely on glycolysis and fatty acid oxidation (FAO) and have low protein synthesis, while OXPHOS and protein synthesis are upregulated during the transition of qNSC to become activated NSC (aNSC) and later IPC [56]. Several groups have studied how knockout of genes encoding mitochondrial proteins affects adult neurogenesis. Conditional deletion in mice of succinate dehydrogenase subunit D *(Sdhd)* in cells of the astrocyte lineage, which includes NSPC of the adult SVZ and DG, impaired differentiation of NPC to neurons and oligodendrocytes without affecting the generation, maintenance, and multi-potency of adult NSC [57]. We found similar phenotypes in mice lacking PTEN-induced kinase 1 (PINK1), mutations in which cause recessive familial Parkinson’s disease [58]. Cultured hippocampal NSPC from PINK1-deficient mice displayed reduced mitochondrial membrane potential (Δψm) and respiration, increased glycolysis, increased apoptosis, and decreased capacity for neuronal differentiation, as shown by fewer DCX^+^ immature neurons and defects in dendritic maturation [58]. In the DG, PINK1 loss delayed the differentiation and reduced dendritic complexity of immature DCX^+^ neurons [58]. Severe mitochondrial dysfunction in NSPC also affected proliferation and cell fate decisions of NSPC, in addition to compromising differentiation of newborn neurons. For example, conditional ablation of mitochondrial transcription factor A (*Tfam*) in NSC of the DG led to a selective reduction of IPC counts and overall reduced proliferation of hippocampal NSPC [59]. Glycolysis- and FAO-related genes were expressed in qNSC and aNSC of the hippocampus but became downregulated in IPC at the same time as expression of mitochondrial genes increased, suggesting that mitochondrial function is already important to promote the transition from aNSC to IPC [59]. In addition, conditional astrocyte lineage-specific deletion of the mitochondrial complex I subunit Ndufs2 during mouse development reduced the proliferation of NSPC isolated from the SVZ and resulted in abnormal cortex development and early postnatal death [60]. Although the average number of proliferating (EdU^+^) NSPC in the DG was also lower in PINK1-deficient mice (in several experiments), this effect did not reach statistical significance [58]. It is likely that conditional *Tfam* knockout causes more severe mitochondrial defects than PINK1 deficiency, because Mito-Park mice with a *Tfam* deletion [61], but not PINK1-deficient mice [62], showed degenerative changes of the dopaminergic system. Based on these studies, it might be proposed that mild mitochondrial dysfunction primarily compromises differentiation of adult-born neurons in the DG due to absolute dependence of differentiated neurons on OXPHOS, while severe defects of mitochondrial bioenergetics (as in *Tfam* and *Ndufs2* knockouts, which may lead to oxidative stress) also impair NSPC proliferation and survival due to the increased dependence of IPC on mitochondrial metabolism. Alternatively, inhibitory effects of mitochondrial deficits on NSPC proliferation may have been partially reversed by compensatory increased glycolysis in NSPC of PINK1-deficient mice, as glycolysis promotes and is sufficient to maintain proliferation of stem cells [39].

### 3.2. Regulation of Adult Neurogenesis by Mitochondrial Dynamics

Mitochondrial dynamics refers to alterations of mitochondrial morphology and subcellular distribution, which is controlled by proteins that promote fission or fragmentation (Drp1, Fis1), fusion (Mfn1/2, Opa1), and transport (Miro, Milton) of mitochondria [34]. Mitochondrial dynamics is required for normal development and cell function, including differentiation and the cell cycle [32,34]. Mutations in mitochondrial fusion genes cause hereditary neuropathies Charcot–Marie–Tooth disease 2A and autosomal–dominant optic atrophy [34], and abnormalities of mitochondrial dynamics are implicated in the pathogenesis of several neurodegenerative disorders, most notably Parkinson’s disease [63].

Studies with Drp1-knockout mice revealed that aberrant mitochondrial dynamics compromises neuronal differentiation and synapse formation during development [64,65]. That mitochondrial dynamics also regulates AHN was demonstrated later by infection of hippocampal NSC with a retrovirus expressing mitochondria-targeted GFP, which allowed permanent labeling of mitochondria in the neurogenic lineage [66]. This revealed that, during differentiation of newborn DG granule neurons, the mitochondrial mass increases dramatically (indicative of mitochondrial biogenesis), coupled with increased mitochondrial fission and distribution of mitochondria from the soma to dendrites of newborn neurons [66]. Voluntary running, which promotes AHN, enhanced these mitochondrial network alterations [66]. Importantly, only a few newborn neurons survived one week after infection of NSC with a retrovirus that co-expressed dominant-negative Drp1 (dnDrp1, which inhibits fission) and mitochondrial GFP, and these neurons had very few mitochondria and failed to extend dendrites into the granule cell layer [66]. Taken together, these results show that mitochondrial biogenesis and fission are essential for the differentiation of neurons by supplying sufficient mitochondria into growing dendrites of adult-born neurons [66]. In another study, failure to distribute mitochondria into dendrites of mice that lack the mitochondrial transport protein Miro1 caused neurodegeneration, corroborating that active transport of mitochondria into dendrites is crucial to sustain dendritic arborization and promote dendritic complexity [67].

PINK1 promotes the degradation of depolarized mitochondria through mitophagy in cooperation with Parkin [68]. We and others have shown that lack of PINK1 alters mitochondrial dynamics, resulting in elongated and swollen mitochondria with cristae degeneration in primary cortical neurons of PINK1-deficient mice [69], *Drosophila* lacking PINK1 [70], and primary hippocampal neurons with RNAi-induced PINK1 knockdown [71]. Shifting the balance of mitochondrial dynamics toward increased fusion in PINK1-deficient neurons [69] may have opposed the mitochondrial fission-dependent dendritic maturation of adult-born DG neurons [66], thereby causing defects in the differentiation of DCX^+^ neurons in mice lacking PINK1 [58].

Khacho et al. showed that inducible knockout of the mitochondrial fusion proteins Mfn1/2 in adult hippocampal NSC reduced the numbers of uncommitted Sox2^+^ NSC and immature DCX^+^ neurons in the DG [72]. Acute knockdown of mitochondrial dynamics proteins Mfn2, OPA-1, and Drp1 in Sox2^+^ NSC showed that aberrant mitochondrial dynamics impacted on NSC self-renewal and fate decisions through changes in ROS signaling, while ATP levels were unaffected [72]. A substantial fraction of adult NSC is derived from a subpopulation of proliferating embryonic NPC [73,74]. To study effects of respiratory chain defects on embryonic neurogenesis and the consequences of mitochondrial dysfunction in embryonic NSPC on AHN later in life, the mitochondrial oxidoreductase AIF was conditionally deleted in vivo in uncommitted NSPC in the early (E9) telencephalon [75]. This led to excessive mitochondrial fission and ROS production at E15.5 associated with impaired self-renewal of Sox2^+^ NSC, enhanced proliferation and impaired cell cycle exit of Tbr2^+^ NPC, and abnormal development of the hippocampus [75]. As a result, adult mice showed depletion of Sox2^+^ NSC in the DG and a complete lack of AHN as manifested by the absence of DCX^+^ immature neurons [75].

Mitochondrial dynamics also regulates neurogenesis in the adult SVZ, which was shown in neurosphere cultures with SVZ-derived adult NSC [76]. In these cultures, mitochondria were localized at the leading process of migratory NSC, and inhibiting Drp1 with Mdivi-1 reduced migration of NSC out of neurospheres and their differentiation to neurons [76].

Collectively, these studies show that mitochondrial fission not only supports differentiation of newborn hippocampal neurons [66], but also regulates early hippocampal NSC fate decisions such as self-renewal and commitment to neuronal differentiation through ROS signaling [72]. On the other hand, excessive fission and ROS production contribute to neurodegeneration and are detrimental for neurogenesis [75,77,78]. Thus, a tightly regulated physiological balance between fission and fusion is crucial for normal neurogenesis.

### 3.3. Regulation of Adult Neurogenesis via Reactive Oxygen Species (ROS) Signaling

Likewise, cellular ROS levels must be tightly controlled. Physiological ROS fulfill important signaling functions in cells and tissues, including during neurogenesis [79,80,81,82]. However, overproduction or impaired detoxification of ROS causes oxidative stress, which contributes to aging, several disorders including neurodegeneration, and impairs neurogenesis [83,84,85]. ROS are produced by mitochondria, NADPH oxidases, and peroxisomes, with the major source being mitochondrial respiration.

Neurogenesis itself transiently produces ROS, as shown in experiments with cultured adult hippocampal NSC, where neuronal differentiation is accompanied by an increase of mitochondrial content and ROS levels [86]. Moreover, markers of oxidized DNA and lipids accumulated in the SGZ of the DG, which was reduced by pharmacological inhibition of neurogenesis [86]. As discussed above, acute experimental disturbances of mitochondrial dynamics in Sox2^+^ NSC affected the decision between NSC self-renewal and differentiation through changes in ROS signaling [72]. Specifically, the transition from a NSC to a committed IPC required mitochondrial fragmentation, which triggered a ROS- and Nrf2-dependent transcriptional program, overall increasing expression of differentiation genes and suppressing self-renewal genes [72]. A more recent study—which FACS-sorted NSPC according to ROS levels and carried out marker and transcriptome analyses on subgroups of NSPC with decreasing ROS levels—unexpectedly showed that the cells with the highest ROS levels were qNSC [87]. Shifts to lower ROS content primed NPC for a subsequent state transition, whereby lower ROS levels correlated with increased expression of proliferation and differentiation genes [87]. In addition, a transient Nox2-dependent burst of ROS production (over the already high levels of basal ROS) promoted exercise-induced recruitment of qNSC for proliferation, but Nox2 was not required for NSPC proliferation under physiological conditions [87]. While these results seemingly contradict earlier results [72], they may not be mutually exclusive, because the transient nature of ROS and ROS signals likely triggers cell transitions without substantially altering ROS levels in the next cell type, especially if the ROS burst also activates anti-oxidative genes [72]. Measuring ROS levels in vivo and at a higher temporal resolution may be necessary to resolve these discrepancies.

In addition to mitochondria, NADPH oxidases (Nox) are the second major source for intracellular ROS [79]. Several Nox enzymes regulate self-renewal, proliferation, and differentiation of NSC during embryonic and adult hippocampal neurogenesis through PI3K/Akt and MAPK/Erk signaling pathways, which has been described in an excellent recent review [79].

Because ROS are central in the regulation of neurogenesis, it is not surprising that environmental or genetic factors, injury, or chronic disease that alter ROS levels can also affect neurogenesis. For example, *Nox2* deletion in mice decreased the population of radial glia-like NSC and neuroblasts under physiological conditions [88]. Despite this, within one week after traumatic brain injury (TBI), Nox2-knockout mice showed increased numbers of neuroblasts, and five weeks after TBI they had significantly more surviving newborn neurons compared to wild type mice [88]. These results suggest that Nox2-derived ROS are necessary to maintain radial glia-like NSC under physiological conditions, but impair the proliferation of NSPC and neuroblasts under pathological conditions such as TBI due to over-activation of Nox2. Likewise, SVZ-derived NPC from mice deficient for the clock gene *Bmal1* showed oxidative stress due to aberrant expression and inactivation of anti-oxidant genes, which compromised NPC proliferation and migration along the rostral migratory stream [89]. Ischemia—which is associated with a surge of oxidative stress—increased the number of newborn neurons in the DG in an Nrf2-dependent manner, and Nrf2 overexpression in cultured hippocampal NSPC enhanced neuronal differentiation, while Nrf2 deficiency exacerbated the detrimental effects of amyloid-β (Aβ) on AHN in mice [90]. Taken together, these studies show that physiological ROS produced by mitochondria and NADPH oxidases critically regulate NSC self-renewal, proliferation, and differentiation in a stage-specific manner, but that excessive and prolonged ROS production (e.g., induced by TBI or Aβ) is detrimental for the survival and maturation of newborn neurons. While the pleiotropic extrinsic factors and intracellular signaling pathways that regulate adult neurogenesis have been described elsewhere [30,31], several key transcription factors and signaling pathways mediate the effects of ROS on adult and embryonic neurogenesis, including Notch and Wnt/β-catenin [72,91], Nrf2 [72,90], p53 [92], PI3K/Akt [92,93,94], and pErk1/2 [95].

## 4. Regulation of Adult Neurogenesis by Autophagy and Lysosomal Degradation

**Figure 2:**
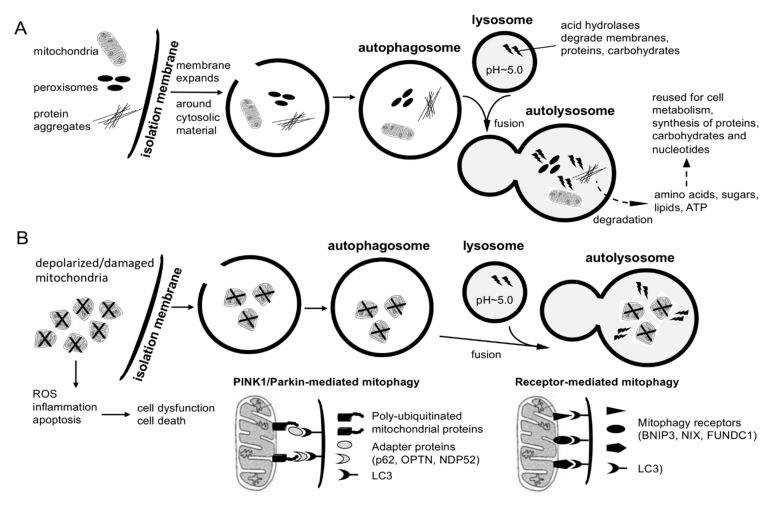
Simplified scheme of autophagy and mitophagy pathways. (**A**) Basal macro-autophagy (autophagy) degrades and recycles cellular contents at steady state. The activity of autophagy is regulated by nutrient (e.g., glucose) and energy (AMP/ATP ratio) availability through PI3K/Akt signaling, mTORC1, and AMPK. Autophagy is induced under conditions of nutrient and energy starvation and by oxidative stress, i.e., conditions that require enhanced recycling of cellular materials to sustain cell metabolism and degradation of oxidized and aggregated proteins to protect cells against stress-induced damage. At the start of autophagy, a double membrane (isolation membrane or phagophore) forms and expands around the cellular material to engulf the material within an autophagosome. The autophagosome fuses with a lysosome that contains hydrolases necessary to degrade the contents within the resulting autolysosome. For details, including stage-specific autophagy regulators, please refer to [96]. (**B**) Mitophagy is a subclass of autophagy that selectively degrades depolarized and damaged mitochondria. There are two mitophagy mechanisms (for details, see [55,68]). In PINK1/Parkin-dependent mitophagy, PINK1 selectively accumulates on depolarized mitochondria (due to import deficiency), and PINK1 and Parkin cooperate to poly-ubiquitinate specific proteins at the surface of depolarized mitochondria. PINK1-phosphorylated polyubiquitin serves a signal for autophagy adapters (p62, OPTN, NDP52) to bind to the damaged mitochondria and target them to autophagosomes via interaction with LC3 of the isolation membrane. In receptor-mediated mitophagy, mitophagy receptors (BNIP3, NIX, FUNDC1) localize to the mitochondrial membrane and directly interact with LC3.

Autophagy is a conserved lysosomal degradation pathway that influences cell survival and metabolism by clearing cells from harmful protein aggregates and damaged organelles, including depolarized mitochondria through mitophagy [55,96]. Autophagy is regulated by nutrient availability and cellular energy status—as well as induced by oxidative stress—and mediated by diverse signaling pathways, including PI3K/Akt, AMPK, and inhibition of mTORC1 [96]. Figure 2 depicts a simplified diagram of autophagy and mitophagy.

The key autophagy genes *Ambra-1* and *Beclin-1* are expressed in the SVZ [97], and autophagic flux was demonstrated in adult hippocampal NSC and their progeny by infecting hippocampal NSC with a retrovirus encoding mCherry-EGFP-LC3 [98]. Targeting genes and miRNAs that regulate autophagy induction and promote autophagic flux, including *Beclin-1*, *Atg5, let-7,* and the *Forkhead Box O* (FoxO) family of transcription factors led to defects of adult NSPC renewal, proliferation, migration, and differentiation in the SVZ [97,99,100] and the DG [98,101,102]. Some of these defects could be rescued by pharmacological induction of autophagy with rapamycin [101] or over-expression of the autophagy inducer Beclin-1 and the transcription factor EB (TFEB), a master regulator of autophagy and lysosomes [99].

FoxO family transcription factors have an essential role in preserving the NSC pool by maintaining a physiological balance between NSC self-renewal and differentiation. Triple-knockout mice deficient for FoxO1, FoxO3, and FoxO4 showed initial hyper-proliferation of NSPC in early adult life, followed by depletion of NSC and severely impaired neurogenesis in the SVZ of adult mice [100]. FoxO3 activity was higher in self-renewing adult NSC than in differentiated progeny, and FoxO3 prevented premature NSC depletion and differentiation by inducing a transcriptional program to preserve NSC quiescence [102]. Using cultured NSC isolated from the SVZ of adult mice, it was shown that FoxO3 directly regulates an autophagy network, binding to and activating about one third of the known autophagy genes as well as several key mitophagy genes, including *Pink1*, *Bnip3,* and *Bnip3L,* thereby increasing autophagic flux [103].

In addition, emerging evidence suggests that mitophagy may regulate both stemness and differentiation of stem cells, possibly in a tissue-specific manner [104]. Indeed, given that mitochondria have essential roles throughout neurogenesis, it is likely that mitophagy—a subclass of autophagy that selectively degrades damaged mitochondria—is also important in this process.

Finally, autophagy is part of a lysosomal degradation pathway, and recent transcriptome profiling has shown that lysosomal function regulates SVZ neurogenesis [105]. Specifically, qNSC in young mice digest accumulating protein aggregates slowly within lysosomes, whereas aNSC rely mostly on proteasomal protein degradation. Activation of qNSC by growth factors increased lysosomal protein degradation, while inhibiting lysosomal activity with bafilomycin-A reduced growth factor-induced protein degradation and activation of qNSC. Age-dependent lysosomal dysfunction also impaired activation of qNSC, which could be corrected by expression of TFEB [105].

Taken together, these studies show that physiological autophagy and lysosomal degradation are crucial for NSC maintenance as well as NPC proliferation, survival, and differentiation to newborn neurons in both the SVZ/OB and the DG.

## 5. Psychological Stress-Induced AHN Defects and Mood Disorders: Contribution of Mitochondrial and Autophagic Dysfunction

In addition to its role in cognitive flexibility [7,8,9,10], AHN provides a buffer against stress-induced depression and anxiety by blunting the brain hypothalamic–pituitary–adrenal (HPA) stress response and/or accelerating recovery from pathologically augmented HPA axis activity [106,107,108,109,110]. Unpredictable averse experiences, or stressors, activate the HPA stress-signaling axis that culminates in the secretion of cortisol in humans and corticosterone in rodents. These glucocorticoids cause physiological and behavioral responses that serve to protect the organism against acute stressors. Importantly, signaling through glucocorticoid receptors expressed in neurons of the hippocampus provides negative feedback inhibition onto the HPA stress response [111,112], resulting in a transient stress response. However, chronic over-activation of the HPA axis—which occurs when a stressor or threat persists—impedes the negative feedback inhibition of the hippocampus and is strongly linked to development of major depression and anxiety disorders [112].

Chronic stress decreases the proliferation of NSC and the survival of newborn neurons in the adult DG [113,114]. Moreover, stress-induced deficits of AHN have been linked with affective dysfunction in animal models across species [113,115,116,117]. While AHN defects per se do not cause affective dysfunction [118,119], several anti-depressants increase AHN, and the beneficial effects of fluoxetine on depressive behavior depended at least in part on neurogenesis [120,121]. Some widely used antidepressants also induced autophagy in hippocampal neurons [122], and stimulation-induced autophagy in the hippocampus was necessary to form new memories and reverted impaired memory in aged mice [123]. Moreover, trehalose and rapamycin—two strong inducers of autophagy—also exerted anti-depressant-like effects in mice, suggesting that autophagy may have mood-stabilizing effects [124,125]. However, these studies did not investigate whether the beneficial effects of autophagy induction on memory and mood involved and required stimulation of AHN.

Mitochondrial dysfunction has been implicated in the development of major depression [126,127] and represents a hallmark of both sporadic and familial forms of PD [63], where depression is a frequent non-motor feature [128]. However, whether mitochondrial defects in the neurogenic niche contribute to, or enhance, stress-induced AHN defects and mood disorders is not known. We addressed this question with PINK1-deficient mice—a model for familial PD—that display abnormal mitochondrial function in hippocampal NSC and impaired AHN in the DG [58]. Specifically, we investigated whether PINK1 deficiency predisposed mice to basal or stress-induced depression. Mitochondrial and AHN defects in PINK1-deficient alone were insufficient to cause depression [58,119]. However, lack of PINK1 exacerbated the stress-induced decline of adult-generated DCX^+^ immature and NeuN^+^ mature neurons in the DG and lowered the threshold for stress-induced depression in mice [119]. Therefore, mitochondrial defects in PD may reduce the resilience to stress-induced depression, a hypothesis worth investigating with additional models. In support of this notion, while chronic stress can cause various mitochondrial deficits [129,130,131,132], neither corticosterone nor mild mitochondrial defects alone affected learning [133]. However, their combination resulted in impaired learning, which showed that stress (corticosterone) and mitochondrial defects synergized to precipitate defects of learning [133]. Taken together, these studies suggest that improving mitochondrial function may attenuate or protect against stress-induced AHN defects and depression.

Although autophagy is necessary for adult neurogenesis under physiological conditions [98,99,101,103], pathologically increased autophagy can be detrimental to AHN and may be involved in development of stress-induced cognitive and affective dysfunction. For example, deletion of the autophagy-promoting gene *Atg7* abrogated corticosterone-induced death of cultured hippocampal NSPC [134]. Moreover, mice with inducible *Atg7* knockout in adult hippocampal NSPC were resilient to chronic stress- and corticosterone-induced deficits of AHN, cognition (spatial memory), and mood (anxiety-like and depressive behaviors) [134]. Therefore, suppressing autophagy may be beneficial for therapeutic intervention in psychological stress-induced disorders [134], although such an approach should be taken cautiously due to the essential function of basal autophagy in most cells and tissues.

## 6. Mitochondrial and AHN Defects in Age and Neurodegenerative Disease: Link to Cognitive and Psychiatric Disturbances

Animal studies support a role of AHN for cognitive flexibility [7,8,9,10]. Accordingly, the age-dependent reduction of AHN may contribute to natural cognitive decline. The involvement of mitochondria in this process is not well understood. However, both aging and age-related neurodegenerative disorders are characterized by mitochondrial abnormalities, including accumulation of mitochondrial DNA (mtDNA) mutations, decreased OXPHOS, and increased mitochondrial ROS production [135,136], all of which impair adult neurogenesis [137,138,139].

With relevance to stem cells, human iPSC derived from tissues of old and young people indicated an age-dependent accumulation of mtDNA damage and a functional mitochondrial decline [140]. Experimentally aging iPSC by continuous propagation also led to abnormal mitochondrial network appearance and mitochondrial gene expression, associated with failure to undergo neuronal differentiation in vitro [141]. Mice with a knock-in of mutant mitochondrial DNA polymerase-γ (*PolgA*-mutator mice) had reduced life span and developed premature aging-related phenotypes due to increased accumulation of mtDNA mutations [142,143]. Cultured adult NSC from *PolgA*-mutator mice showed impaired self-renewal and increased mtDNA mutations and ROS production, which led to a reduction of NSC in the SVZ of old *PolgA*-mutator mice [137]. NSPC from the forebrain of aged wildtype (normal) mice also revealed lower mitochondrial content, respiration rates, and ATP synthase levels compared to young animals [144]. Taken together, these studies show that defects of mitochondria contribute to aging-related impairments of AHN and, therefore, likely also age-related cognitive decline, although the latter notion requires additional experimental support for confirmation.

Mitochondrial dysfunction is also a hallmark of many neurodegenerative disorders including PD [63] and Alzheimer’s disease (AD) [145,146], and it has been suggested that impaired AHN may contribute to cognitive and psychiatric disturbances in PD [147,148] and AD [149,150,151,152]. While it is difficult to reliably assess neurogenesis in post-mortem brains and few such studies have been carried out to date, reduced numbers of proliferating cells in the SVZ and neural precursor cells in the DG and the OB were reported in post-mortem brains of individuals with PD [153]. This finding was replicated in mice by experimental dopamine depletion, which led to the suggestion that dopamine regulates adult SVZ neurogenesis [153]. However, a later study found no difference in the number and proliferation of NSPC between PD subjects and age- and sex-matched controls, nor was SVZ neurogenesis abnormal in five cases with incidental Lewy body disease [154]. Deficits of AHN were also reported in post-mortem brains of AD patients and individuals with mild cognitive impairment (MCI) [151,152,155]. Increased expression of bone morphogenetic protein 6 in the hippocampus of AD patients correlated with reduced numbers of Sox2^+^ NPC and DCX^+^ immature neurons [155]. In a study with 45 patients who died of AD between 52 and 97 years, stereology revealed abnormalities of AHN already in the brain of early-stage AD patients, and the numbers and maturation of DCX^+^ neurons declined progressively with increasing neuropathology (Braak stages) of AD [151]. Nestin^+^/Sox2^+^ NPC and DCX^+^ neuroblasts persisted in the brain of old people, and their numbers were reduced in brains from patients with both MCI and AD when compared to age-matched controls [152]. Cognitive performance in MCI patients correlated positively with the number of DCX^+^ neuroblasts, suggesting an association of AHN with cognition [152].

A link between impaired AHN and cognitive and psychiatric disturbances in neurodegeneration is further corroborated by studies in models of PD [58,119,156,157,158,159,160] and AD [161,162,163,164,165,166]. Although the mechanisms responsible for impaired AHN are pleiotropic, in the case of PINK1-deficient mice, mitochondrial defects in NSPC have been linked to impaired dendritic maturation of DCX^+^ neurons in the DG [58]. In addition, both α-Synuclein and amyloid-β (Aβ)—which form neurotoxic protein aggregates in PD and AD—impair mitochondrial respiration [145,167], suggesting that in transgenic models overexpressing these pathogenic proteins mitochondrial defects also contributed to abnormal AHN [157,158,165,166]. Finally, expression of Miro2—which regulates mitochondrial transport and dynamics and is degraded in a PINK1/Parkin-dependent manner upon induction of mitophagy [168,169], was decreased in Nestin-positive cells of the hippocampus in the 3xTg mouse model of AD [77]. Suppressing Miro2 (by Miro2-siRNA or an Miro2-targeting miRNA) in cultured adult hippocampus-derived NSC of normal mice led to excessive mitochondrial fission, ROS production, and autophagic cell death of NSPC, which was rescued by adenoviral Miro2 over-expression and the Drp1/fission inhibitor Mdivi-1 [77]. Collectively, these studies support the notion that mitochondria-related abnormalities of AHN contribute to cognitive and psychiatric disturbances in neurodegenerative illnesses.

## 7. Targeting Mitochondria to Counteract AHN and Cognitive Defects in Old Age and Disease

Given the critical function of mitochondria throughout adult neurogenesis, targeting mitochondria to improve AHN may be a promising strategy to mitigate age-related cognitive decline and cognitive and psychiatric dysfunctions in neurodegenerative diseases.

Metformin—an FDA-approved drug used for the treatment of type-2 diabetes [170]—shows promise in enhancing adult neurogenesis [171]. Metformin improved mitochondrial function and was neuroprotective in a model of sporadic PD [172], and it enhanced adult neurogenesis and cognition in models of neuronal dysfunction induced by type-2 diabetes and ischemia [173,174,175]. Metformin promotes mitochondrial biogenesis through induction of PGC-1α, a transcriptional coactivator that coordinately induces expression of many mitochondrial metabolism and anti-oxidant genes [172,176,177]. Metformin also stimulates autophagy via AMPK [96]. However, in some studies, the effects of metformin on neurogenesis and cognition were sex-specific [178,179], and in one AD model metformin exacerbated neuropathology and disease phenotypes [180]. Piracetam, which improves mitochondrial bioenergetics and inhibits mitochondrial permeability transition [181], ameliorated aging-associated defects of cultured hippocampal NSPC and increased AHN in old mice [59]. However, piracetam failed to restore AHN in old mice to levels observed in young mice, and whether piracetam-induced AHN improved cognitive performance in aged mice was not investigated [59].

Certain dietary supplements and lifestyle choices have considerable potential to attenuate neuronal dysfunction and enhance neurogenesis through mitochondrial improvement [45,182]. NAD^+^ acts as a coenzyme in many redox reactions and a co-substrate for metabolism-regulating enzymes including the sirtuins and PARP [182,183]. Sirtuins are deacetylases that mediate the effects of caloric restriction on longevity and adapt cellular metabolism to nutrient availability [184]. Mitochondria-localized sirtuins regulate mitochondrial respiration and signaling, as well as mitochondrial biogenesis through PGC-1α [182,184,185]. Supplementation of NAD^+^, whose levels decline during aging [183,186], improved mitochondrial function [183,187,188,189] and cognitive performance [190] in animal models of AD and PD. In contrast, NSPC-specific ablation of the rate-limiting enzyme for NAD^+^ synthesis (Nampt) reduced proliferation of NSPC [186], and conditional deletion of *Nampt* in forebrain excitatory neurons caused hippocampal and cortical atrophy associated with multiple behavioral and cognitive defects [191]. Exercise also exerts beneficial effects on brain mitochondrial function [192,193,194,195]. Exercise-induced AHN improved cognitive function in models of intellectual disability and Alzheimer’s disease [161,196,197], as shown previously in normal mice [17,114,198,199,200]. Overall, these studies suggest NAD^+^ supplementation, activation of specific sirtuins, and exercise as promising strategies to counteract age- and disease-related defects of AHN and cognition.

Influencing the activity of certain signaling pathways and transcription factors that impact on mitochondria may also be explored to enhance adult neurogenesis. The Wnt signaling pathway is important for cell polarity and differentiation, and Wnt-5a has been shown to regulate mitochondrial dynamics and calcium homeostasis in hippocampal neurons [201]. Overexpression of the pro-neural transcription factor Neurod1 in adult-born neurons improved mitochondrial biogenesis and respiration and reversed defects of dendritic growth and spine formation in a mouse model of AD [202]. Finally, an inhibitor of PDE7—by targeting the cAMP/CREB pathway—reversed Aβ-induced mitochondrial defects, improved AHN, and rescued cognitive impairments in the APP/PS1 model of AD [203].

## 8. Summary and Concluding Remarks

**Figure 3:**
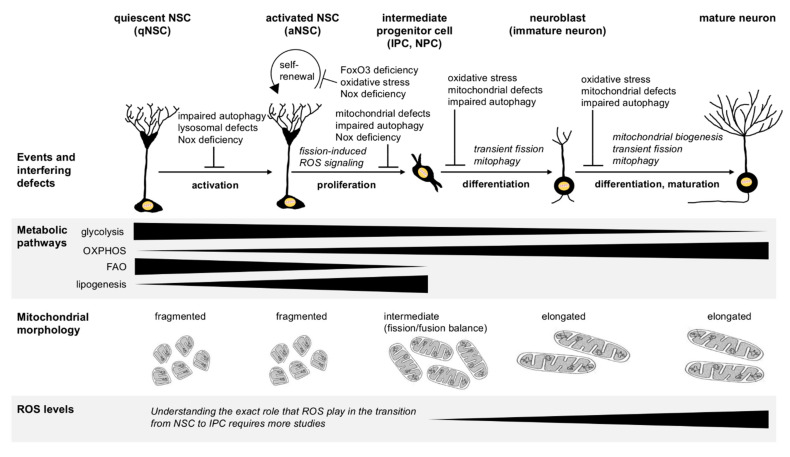
Stage-specific metabolic pathways, mitochondrial dynamics, and ROS regulate cell transition and progression during adult neurogenesis. Neural stem cells (NSC) rely on glycolysis and fatty acid oxidation (FAO) for stemness and self-renewal. An increase of mitochondrial oxidative phosphorylation (OXPHOS) at the expense of FAO and upregulation of de novo lipogenesis in the cytoplasm promote proliferation of NSC and progression toward intermediate progenitor cells (IPC). As IPC begin to differentiate to neuroblasts and further progress to become mature newborn neurons, OXPHOS is crucial to supply the growing cells with sufficient energy. In this phase, mitochondrial biogenesis, fission, and transport are essential to increase mitochondrial content and distribute newly generated mitochondria into the growing and distal branches of maturing dendrites. However, because fragmented mitochondria produce less ATP and fission increases ROS, fission must be transient and followed by fusion. Overall, the mitochondrial morphology changes from fragmented to increasingly elongated during neurogenesis consistent with the progressive reliance of cells on OXPHOS. Reactive oxygen species (ROS) generated by several different NADPH oxidases (Nox proteins) regulate the transition between qNSC and aNSC, NSC renewal, and NSPC proliferation, which has been reviewed elsewhere [79]. In addition, ROS/Nrf2-dependent signaling triggered by mitochondrial fission promotes the transition from aNSC to differentiation-committed IPC. However, the exact role of ROS in this step remains somewhat ambiguous. For more details, please refer to the main text.

Changes of mitochondrial metabolism and structure are not merely secondary adaptations to variable energy demands of different cells along the neurogenic lineage. Instead, mitochondria act as signaling platforms that actively control all stages of neurogenesis, from NSPC fate decisions and proliferation to neuronal differentiation and synaptic integration (Figure 3). The mitochondria-orchestrated program involves stage-specific changes of mitochondrial respiration, biogenesis, dynamics, and ROS production. Mitochondrial fission and ROS—along with ROS produced by NADPH oxidases—tune the activity of specific transcriptional programs (e.g., Nrf2) and cytosolic signaling proteins (e.g., PI3K/Akt, Erk1/2), which is crucial for the earliest NSC fate decisions and transitions from qNSC to IPC by altering the balance between proliferation and differentiation genes, among many others. Once neuroblasts start to differentiate, a coordinated increase of mitochondrial biogenesis, fission, and transport ensures that the growing dendrites of newborn neurons are supplied with sufficient mitochondria, which is crucial for dendritic maturation and the survival of newborn neurons. In addition, autophagy—and in particular the selective degradation of damaged mitochondria through mitophagy—is necessary throughout neurogenesis to maintain a healthy cellular mitochondrial network and ROS balance.

Lifestyle choices that improve mitochondrial function and increase neurogenesis – such as eating food rich in polyphenols and polyunsaturated fatty acids, regular moderate exercise, or select dietary supplements—all show considerable promise to attenuate age- and disease-related cognitive decline. Beyond that, screening for new drugs that selectively augment mitochondrial function and mitophagy (as opposed to autophagy in general)—perhaps by targeting specific mitochondrial respiratory complexes, chaperones, or mitophagy proteins—may lead to improved treatments for cognitive dysfunction in humans. It will be crucial to test effects of new drug candidates on mitochondrial function, neurogenesis, and cognition both in cell culture (i.e., in vitro differentiation systems) and relevant animal models of disease, including their long-term safety and efficacy in vivo. Finally, drug-induced stimulation of mitochondrial metabolism and mitophagy should be moderate—and if possible combined with increasing anti-oxidant responses—as excessive activation of these pathways can cause oxidative stress and mitophagic cell death [204,205]. In this sense, targeting the PGC-1α master regulator that coordinately increases mitochondrial biogenesis and anti-oxidant gene expression may be particularly promising [206].

Finally, the recognition that metabolism takes center stage in the regulation of NSC function and adult neurogenesis is expected to improve the generation and amplification of patient-derived (autologous, non-immunogenic) iPSC and iPSC-derived neuroblasts and neurons for transplantation into areas of neurodegeneration or the injured brain [207,208,209,210,211,212]. Conditions that favor glycolysis and fatty acid metabolism should facilitate de-differentiation of adult somatic cells to iPCS and amplification of iPSC, while conditions that suppress glycolysis and induce mitochondrial metabolism should promote efficient differentiation of iPSC to neurons. Increasing the yield of patient-derived iPSC should also facilitate correction of genetic defects by CRISPR/Cas9-mediated gene editing and the characterization of gene-corrected iPSC or iPSC-derived neurons before transplantation.
